# Mechanistic Studies of the Calcium-Dependent Antibiotics via Cofactor Engineering

**DOI:** 10.1021/acs.jnatprod.5c01440

**Published:** 2026-01-15

**Authors:** Shao-Lun Chiou, Yu-Chi Chang, Ya-Rong Chen, Thomas Ma, John Chu

**Affiliations:** ⊥ Department of Chemistry, 33561 National Taiwan University, No. 1 Section 4, Roosevelt Road, Taipei 10617, Taiwan; § Center for Emerging Material and Advanced Devices, National Taiwan University, Taipei 10617, Taiwan

## Abstract

The defining feature of calcium-dependent antibiotics (CDAs) is that they require the presence of calcium cation (Ca­(II)) as a cofactor to exert antibacterial activity. We recently showed that substituting two key aspartic acids (Asp) with serine (Ser) in laspartomycin C (LspC) converts it from a CDA into a boron-dependent antibiotic (BDA). This synthetic analog (termed **B1**) no longer depends on Ca­(II) and requires only 10 μM of phenylboronic acid (PBA) to become fully active. Such a calcium-to-boron dependence conversion provides a new entry point to study the mechanistic details of the cofactor dependence of CDAs, a rare phenomenon among bioactive small molecules. Herein, we show that electron withdrawing substituents on PBA enhance the antibacterial activity of **B1**. The friulimicin and daptomycin synthetic analogs with the same Asp-to-Ser substitution were inactive, whereas the CDA4b synthetic analog exhibited dual cofactor dependence. CDA4b was fully activated when both Ca­(II) and PBA were present and was 4-fold less potent in the presence of only one or the other. These findings suggest that not only do CDAs often have distinct cellular targets, the way they are activated by Ca­(II) are also different. Such mechanistic diversity underscores the strong potential of CDAs in drug development.

## Graphical abstract

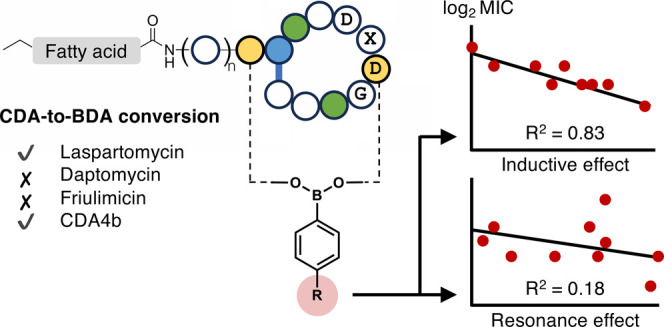

The first calcium-dependent antibiotic (CDA) amphomycin was discovered in 1953.^1^ Many more were identified since then, and the CDA family currently has over 10 members. All known CDAs suppress bacterial growth by disrupting cell wall biosynthesis.^2,3^ Some sequester key biosynthetic intermediates, e.g., cardiolipin, lipid II, undecaprenyl phosphate (C55P),^3–5^ and some alter the curvature of the cell envelope via the formation of a multipartite complex.^6,7^ Since the cell wall biosynthesis pathway is absent in humans, scientists have long been interested in CDAs as drug candidates and are constantly searching for new ones. The classic culture extract screening approach had identified CDAs such as the “calcium-dependent antibiotics” (homonymous to its family name, henceforth referred to as CDAx),^8^ laspartomycin (LspC),^9^ A54145,^10^ daptomycin (Dap),^6^ etc. In recent years, culture-independent metagenomic approaches led to the discovery of malacidin,^7^ cadaside,^11^ and ambocidin.^3^

While antibiotic families are typically categorized based on a shared mechanism of action (MOA) due to the presence of a common core structure, this is not the case for CDAs, as most of them have their own distinct targets. The defining feature of this antibiotic family is instead the fact that they all require calcium cation (Ca­(II)) as a cofactor to exert antibacterial activity. However, for most CDAs the mechanistic details of their calcium dependence remain elusive. Furthermore, human physiological Ca­(II) concentration (1.0 to 1.5 mM) is not enough to fully activate most CDAs.^12^ Understanding the molecular basis of calcium dependence is therefore of both scientific interest and practical importance. In this context, we engineered laspartomycin C (LspC) and converted it from a CDA to a boron-dependent antibiotic (BDA, Figure 1), termed **B1**, by replacing two aspartic acid residues (Asp1 and Asp7) in LspC with serine (Ser).^13^ We showed that while LspC requires approximately 5 mM Ca­(II) to exert maximum potency, the synthetic analogue **B1** no longer requires Ca­(II) and can be fully activated with only 10 μM of phenylboronic acid (PBA). The MOA of **B1** remained the same as LspC, i.e., sequestering the key bacterial cell wall biosynthesis intermediate C55P. Notably, **B1** is effective against prominent Gram-positive bacterial pathogens, e.g., methicillin-resistant *Staphylococcus aureus* (MRSA) and vancomycin-resistant *Enterococcus faecium* (VRE).

**Figure 1:**
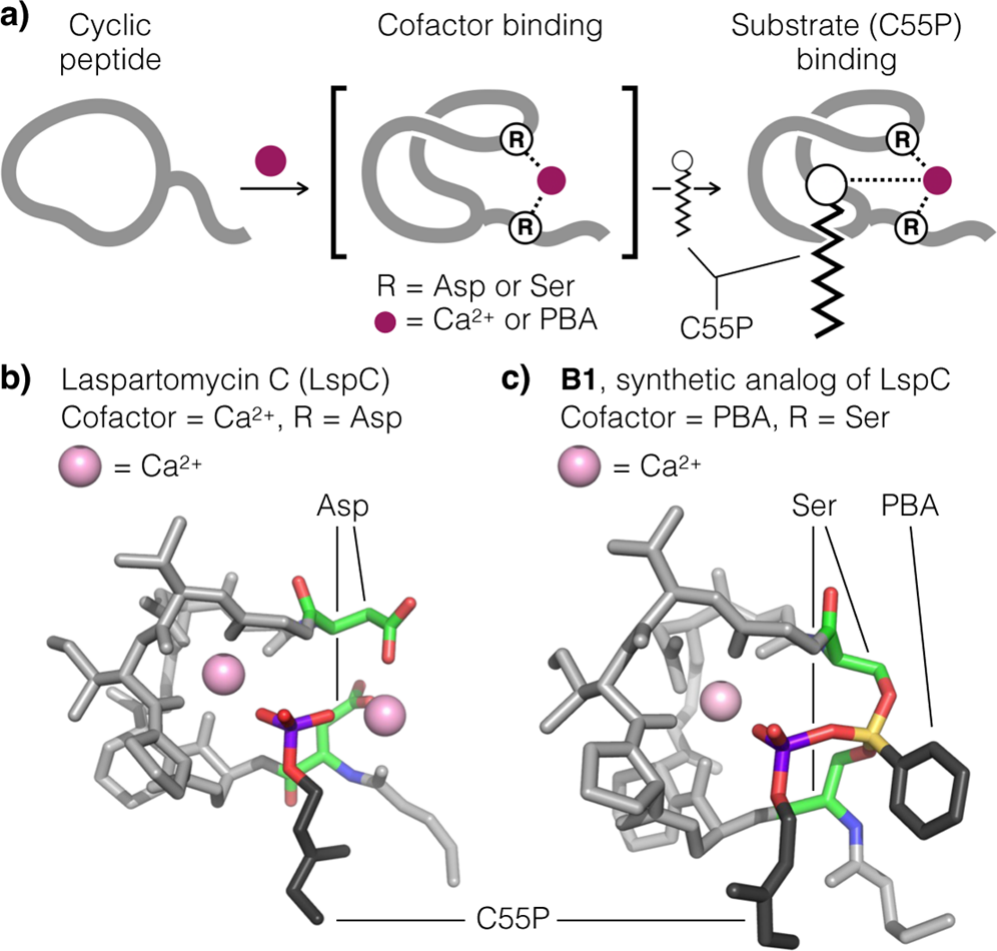
a) A cofactor (Ca­(II) or PBA, pink sphere) induces LspC to fold and adopt a conformation poised to bind undecaprenyl phosphate (C55P). (b) The native LspC (R=Asp) requires Ca­(II) for activation. Shown here is the ternary complex LspC/Ca­(II)/C10P obtained via X-ray crystallography (PDB: 5O0Z). (c) The synthetic analog **B1** (R=Ser) requires PBA for activation. Shown here is the quaternary complex **B1**/PBA/C10P/Ca­(II) obtained via MD simulation.

The finding described above is significant in two ways. First, a BDA is more broadly applicable, as it is no longer limited to locations with high concentrations of Ca­(II). Second, it provides a new entry point in studying the calcium dependence mechanism of CDAs. The current study focuses on the latter aspect. We first tested a panel of para-substituted PBAs on **B1** and showed that electron withdrawing groups enhanced its antibacterial activity through inductive effect. In addition, when the Asp-to-Ser substitution design in LspC was applied to three other members of the CDA family (friulimicin B (FruB), Dap, and CDA4b), the resulting synthetic analogs did not show a comparable calcium-to-boron cofactor dependence conversion. Two analogs were inactive, and the third required the presence of both cofactors for activity. Such divergent outcomes suggest that CDAs not only act on distinct cellular targets, but also differ in the way they are activated by Ca­(II).

## Results and Discussion

### Cofactor Properties Affect Antibacterial Activity

We recently reported **B1**, a synthetic analog of LspC and showed that it is an antibiotic that requires PBA as its cofactor.^13^ Detailed characterization by MS, NMR, MD simulation, and mobility shift assays suggests that both LspC and **B1** suppress bacterial growth by sequestering undecaprenyl phosphate (C55P), a key cell wall biosynthesis intermediate. While LspC cannot be activated by metal cations other than Ca­(II), various substituents can be installed onto the benzene ring of PBA, offering a unique opportunity to study how the antibacterial activity of **B1** changes in response to fine-tuning the physical and chemical properties of its cofactor.

We tested the antibacterial activity of **B1** in the presence of 11 different PBAs with para-position substitutions, including both electron-withdrawing and electron-donating groups (Table S1). All arylboronic acids were supplemented at a fixed concentration (100 μM) and the extent of **B1** activation was assessed by minimum inhibitory concentrations (MICs) against *S. aureus*, which ranged from 4 to 32 μg/mL (Figure 2a). We then analyzed the effect of para-substitution by plotting Log_2_ MIC vs the Hammett constant (σ_p_), which is a parameter that has been used to investigate the substituent effect on aromatic rings (Table S2).^14^ Note that some CDAs show a weak tendency to dimerize or oligomerize in the presence of Ca­(II) and may be sensitive to steric hindrance around the cofactor.^15,16^ We therefore chose to exclude the unsubstituted PBA (R=H) from our analysis. Because more potent antibiotics display smaller MIC values, the moderate negative correlation in this plot suggests that electron withdrawing substituents on PBA enhance the antibacterial activity of **B1** (*R* ^2^ = 0.48, Figure 2b). When the inductive and resonance contributions were plotted separately using the modified Swain–Lupton constants *F* and *R*, respectively,^14^ the former has a much stronger correlation than the latter (*R* ^2^ = 0.83 vs 0.18, Figure 2c,d), suggesting that substituents on PBA affect **B1** activation mainly through inductive effects.

**Figure 2:**
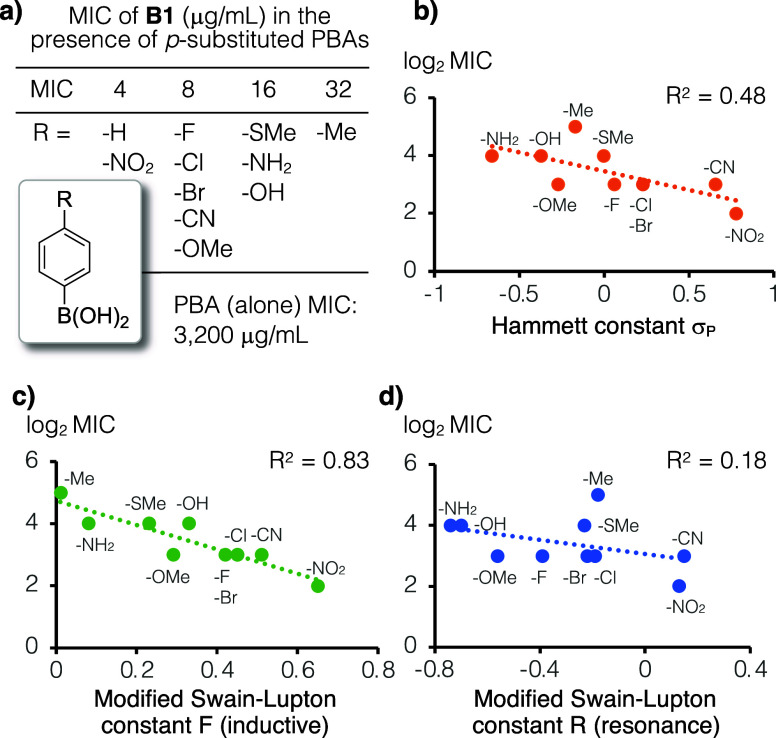
a) Mixtures of **B1** with various para-substituted PBAs (100 μM) were tested against *S. aureus* ATCC 29213. The synthetic analog **B1** by itself is inactive even at the highest tested concentration (128 μg/mL); the MIC of PBA alone is 3,200 μg/mL which is approximately 40-fold higher than what was used in this work. (b) The log_2_ MIC (μg/mL) vs Hammett constants (σ_P_) plot shows a moderately negative correlation, suggesting that PBAs with more electron withdrawing substituents at the para-position are more effective activators of **B1** antibacterial activity. (c,d) The modified Swain–Lupton constants *F* (inductive effect, *R* ^2^ = 0.83) shows a much stronger correlation to antibacterial activity than that of constant *R* (resonance effect, *R* ^2^ = 0.18).

### Identify Commonalities and Differences among CDAs

To identify conserved features, we collected and aligned the sequences of all known CDAs (Figure S1). All CDAs are branched cyclic peptides with one to three exocyclic residues and an N-terminal fatty acyl chain. In this work, we set the branch point as the zeroth position (P0) and assigned positive and negative numbers to residues inside and outside the macrocycle, respectively. Based on this numbering system, common to all CDAs is the DXDG motif that spans P3 to P6, wherein X indicates any amino acid or the absence thereof (Figure 3a). The DXDG motif was once regarded as the sole feature that defines a CDA. However, the fact that LspC can be converted to a BDA (**B1**) by replacing Asp with Ser at P-1 and P5 suggests that there are additional factors at play. Furthermore, the biosynthetic machinery of CDAs (nonribosomal peptide synthetases) can work with both d- and l-amino acids. Residues at P-1 and P5 are invariably l-amino acids, and residues at P1 and P7 are invariably d-amino acids (or glycine). Finally, the residue at P5 is always Asp, and the residue at P-1 is, in most cases, also Asp.

**Figure 3:**
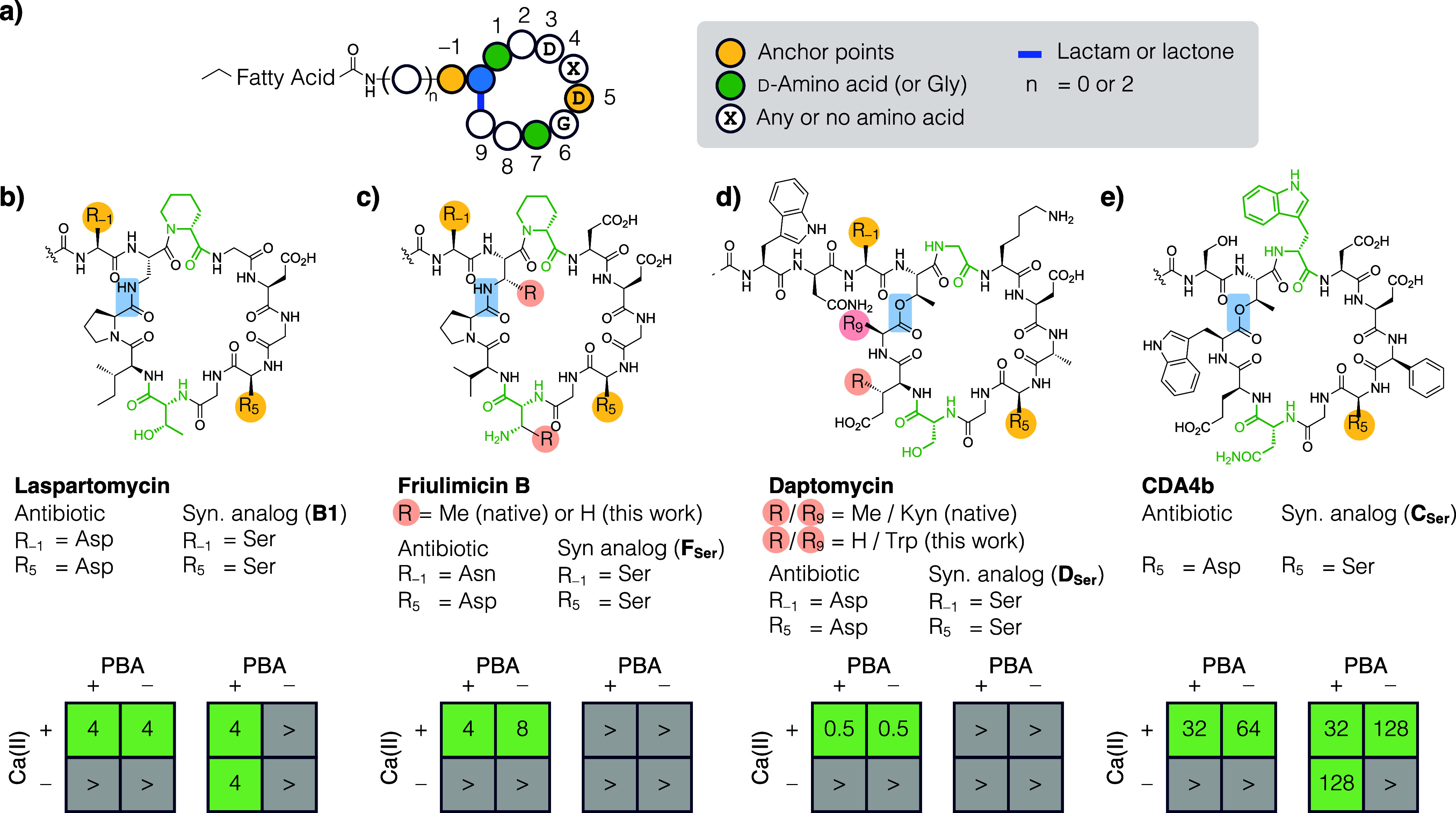
a) All CDAs are branched cyclic peptides with one or three exocyclic residues and an N-terminal fatty acyl chain. The C-terminus carboxylate condenses with a side-chain nucleophile to form a macrolactam (LspC and FruB) or a macrolactone (Dap and CDA4b). In LspC, Ca­(II) coordinates the side-chain carboxylate of Asp at P-1 and P5 to promote a folded conformation (see Figure 1a). This conformation is poised to bind the cell wall biosynthesis intermediate C55P. Note that to streamline peptide synthesis, certain noncanonical amino acids (pink circles) were replaced with structurally similar building blocks that are commercially available. (b) We reported previously that Asp-to-Ser substitutions at P-1 and P5 in LspC (termed the anchor points) resulted in a conversion of cofactor dependence from calcium (Ca­(II)) to boron (PBA). (c–e) This Ser replacement design was tested on three more CDAs, wherein the antibacterial activity of the native antibiotic and the synthetic analog with Ser substitution(s) were assessed in the presence and absence of both Ca­(II) and PBA. The results are presented in a two-by-two grid format. Antibacterially active conditions were shown in green and inactive ones in gray. MIC numbers are reported in μg/mL against *S. aureus* ATCC 29213; “>” indicates no inhibition at the highest tested concentration (128 μg/mL). The synthetic analogs of FruB (**F**_**Ser**_, **c**) and Dap (**D**_**Ser**_, **d**) were inactive, and both failed to recapitulate the cofactor dependence conversion seen in LspC. Interestingly, Ca­(II) and PBA work in synergy to activate the synthetic analog of CDA4b (**C**_**Ser**_, **e**), which is 4-fold less potent when only one of the two cofactors were added.

Crystal structure of the LspC/Ca­(II)/C10P ternary complex suggests that Ca­(II) activates LspC by coordinating the side-chain carboxylate of two key Asp residues to promote a folded conformation.^17^ Specifically, the two Asp residues that are involved are located at P-1 and P5 in LspC, and **B1** differs from LspC only by two Asp-to-Ser substitutions at these positions (Figure 3a). LspC is poised to bind C55P upon Ca­(II) promoted folding, and **B1** likely adopts a similar folded conformation upon PBA mediated boronic ester formation that brings the side-chain hydroxyl groups of Ser at P-1 and P5 into proximity. With this information at hand, we went on to examine whether Asp-to-Ser replacements in CDAs other than LspC would also result in a similar cofactor dependence conversion from calcium to boron.

### The CDA-to-BDA Conversion is Only Applicable to LspC; the Synthetic Analog of CDA4b Shows Dual Cofactor Dependence

Three CDAs (FruB, Dap, and CDA4b) were chosen strategically for this study to include ones with maximal to minimal LspC structural similarity. FruB is highly similar to LspC, and both suppress bacterial growth by sequestering C55P.^5,16^ In contrast, aside from features common to all members of the CDA family (see above), Dap and CDA4b show little structural similarities to LspC or to each other. They have distinct targets, i.e., CDA4b binds to cardiolipin and daptomycin interacts with phosphatidylglycerol and lipid II.^18,19^ The exocyclic structures are also different among these CDAs, wherein Dap is the only one with three exocyclic residues, and the others (LspC, FruB, and CDA4b) have only one residue. As with most CDAs, Dap and LspC both have Asp at P-1, whereas FruB and CDA4b are exceptions to this rule and have asparagine (Asn) and Ser at this position, respectively. It should be noted that Asn and Ser in principle can also coordinate to metal cations, albeit with weaker affinity.^20^

For each CDA, we prepared the native antibiotic and the synthetic analogue, wherein residues at P-1 and P5 were replaced with Ser. Peptide synthesis was based on standard solid-phase Fmoc chemistry; slight modifications and additional steps for constructing the macrocycle were described in the Supporting Information (Schemes S1–S3) and performed in accordance to literature precedents.^16,18,21^ Note that to streamline peptide synthesis certain noncanonical amino acids were replaced with structurally similar building blocks that are commercially available. For example, β-methylaspartic acid at P2 of FruB was replaced with Asp, kynurenine at P9 of Dap was replaced with tryptophan, etc. (R groups with pink circles in Figure 3c,d). In this article, the “native” antibiotic refers to a CDA obtained via this streamlined synthetic procedure. All of these noncanonical amino acid replacements have been reported previously; the potency of these antibiotics was reduced to some extent, but their MOA remained the same.^16,18,22,23^ The calcium dependence of the native antibiotics was first evaluated. We determined that 1.25, 5.0, and 16 mM Ca­(II) was sufficient to fully activate Dap, FruB, and CDA4b, respectively, and in subsequent experiments, these amounts of Ca­(II) would be added to the medium for each CDA (if any). Antibacterial assays were performed against *S. aureus* in the presence and absence of Ca­(II) and PBA, and the results were presented in a two-by-two grid format.

LspC was our prototypical case of a calcium-to-boron-dependent conversion (Figure 3b). Specifically, it was fully active in the presence of Ca­(II) irrespective of PBA (MIC 4 μg/mL), and its synthetic analogue **B1** was equally active in the presence of PBA irrespective of Ca­(II). As expected, native FruB, Dap, and CDA4b were all able to suppress bacterial growth in the presence of Ca­(II) (MIC = 4, 0.5, and 32 μg/mL, respectively), irrespective of the presence of PBA. However, under all conditions, the Ser replacement synthetic analogs of FruB (**F**_**Ser**_) and Dap (**D**_**Ser**_) failed to show antibacterial activity even at the highest tested concentration (128 μg/mL). Since the amino acid at P-1 in CDA4b is already Ser, its synthetic analogue (**C**_**Ser**_) differs from the native structure by only a single Ser replacement at P5. Interestingly, **C**_**Ser**_ was just as potent as the native CDA4b when the medium was supplemented with both Ca­(II) and PBA (MIC 32 μg/mL), but 4-fold less active when only Ca­(II) or PBA was added (MIC 128 μg/mL).

### Compare and Contrast the Synthetic Analogs with CDAs to Learn About the Mechanism of Cofactor Dependence

We previously reported that LspC can be converted from a CDA to BDA when the two Asp at P-1 and P5 were replaced with Ser.^13^ The antibacterial activity of the resulting analogue (**B1**) was fully activated by PBA irrespective of Ca­(II) and is just as potent as the original antibiotic (4 μg/mL). The ability of a panel of 11 para-substituted PBA to activate **B1** was tested in this work, and our analysis showed that the strength of electron withdrawing groups on PBA correlates with the potency of **B1** through an inductive effect. This trend is in line with boronic ester formation, wherein boronic acids with lower p*K*_a_ tend to show higher equilibrium constants for this reaction. These observations are also in line with the notion that PBA promotes the folding of **B1** via boronic ester formation with its Ser at P-1 and P5 (Figure 1a).

The MIC of Dap and FruB against *S. aureus* are 0.5 and 4 μg/mL, respectively, whereas their synthetic analogs were inactive even at the highest tested concentration (MIC > 128 μg/mL). While our attempt to convert Dap into a BDA failed, we expected the Ser replacement design that worked on LspC to be applicable to FruB, as they target the same biosynthetic intermediate (C55P) and have highly similar structures.^16^ Surprisingly, the FruB synthetic analogue with Ser replacements (**F**_**Ser**_) also failed to suppress bacterial growth under all conditions tested. This observation suggests that the Ca­(II) activation mechanism in FruB and LspC have subtle differences and may help explain the paradoxical observation by Martin and co-workers.^16,24,25^ They reported that FruB and LspC form very similar ternary complexes to C55P, yet these two antibiotics do not show cross resistance.

CDA4b presents an intriguing case, wherein Ca­(II) and PBA were both needed to fully activate its Ser replacement synthetic analog (**C**_**Ser**_). The MIC of CDA4b is 32 μg/mL, and **C**_**Ser**_ was just as active as the native antibiotic when both Ca­(II) and PBA were supplemented in the medium but was 4-fold less potent in the presence of only one of the two cofactors (128 μg/mL). These observations suggest that native CDA4b likely has two separate yet equally important Ca­(II) binding sites, one of which was converted to a PBA site in the synthetic analogue **C**_**Ser**_, and these two sites work in synergy to turn on its antibacterial activity. This notion corroborated what Taylor and co-workers recently reported, that is, CDA4b goes through two conformational changes upon the addition of Ca­(II).^18^

## Conclusion

Most antibiotics are classified according to a shared MOA, typically arising from the presence of a common core structure. For example, all penicillins contain a β-lactam moiety and act as a covalent inhibitor of enzymes that catalyze peptidoglycan cross-linking. CDAs are an exception to this rule of antibiotics classification. They act on distinct cellular targets and do not show cross resistance. Instead, they are defined by the requirement for Ca­(II) as a cofactor to exert antibacterial activity. Two key Asp-to-Ser substitutions in LspC converted it from a CDA to a BDA, whereas analogous Asp-to-Ser substitutions in Dap and FruB resulted in synthetic analogs (**D**_**Ser**_ and **F**_**Ser**_) that are completely inactive. Interestingly, Ca­(II) and PBA acted in synergy to activate the synthetic analogue of CDA4b (**C**_**Ser**_). Together, these findings underscore the fact that not only do their cellular targets differ, the way CDAs are activated by Ca­(II) are also different from each other.

Scientists have long been interested in the therapeutic potential of CDAs. For example, Dap is an antibiotic approved for clinical use and was designated by the World Health Organization as a critically important part of human medicine. FruB advanced to phase I clinical trial and was terminated due to poor pharmacokinetics.^26^ Our results suggested that CDAs may not warrant classification into a single antibiotic family. Cross resistance is less likely to emerge as bacteria would need to acquire resistance independently to each mechanistically orthogonal CDAs. This feature bodes well for their future prospects in drug development.

## Experimental Section

### Chemicals, Instruments, and General Methods

Solvents and reagents are of ACS grade (or higher) and used as is. All para-substituted PBAs were purchased from vendors listed in Table S1. Lysogeny broth (LB) was purchased from BioShop Canada. Peptides were purified using a C18 semipreparative column (HYPER GLD AQ PREP, 5 μm, 250 × 10 mm, ThermoFisher Scientific) by HPLC (model 600 pump and controller equipped with a 996 UV detector, Waters) using a two-solvent gradient system. Solvent A and B are water and acetonitrile, respectively; both solvents are supplemented with formic acid (0.1% v/v). All compounds are ≥95% pure based on peak integration. Mobility shift assays were performed on a TLC Silica Gel 60 F254 (Merck). High resolution mass spectra of synthetic peptides were acquired using ESI-TOF (microTOF-QII, Bruker). All NMR spectra were acquired on a 400 MHz instrument (AVIII 400, Bruker).

### Peptide Synthesis

The Initiator + Alstra (Biotage) was used for microwave-assisted solid-phase peptide synthesis according to the instructions provided by the manufacturer. Each cycle contains coupling, deblocking, and dimethylformamide (DMF) washes after each step. In a typical coupling step, amino acid building blocks (5 equiv, 0.45 M in DMF), diisopropylcarbodiimide (5 equiv, 0.5 M in DMF), and 1-hydroxybenzotriazole (5 equiv, 0.5 M in DMF) were added to the resin. The coupling reaction was heated by microwave to 75 °C with constant physical oscillation for 5 min. The Fmoc protecting group was removed by performing two rounds of deblocking using 20% piperidine in DMF at room temperature for 10 min. Detailed synthetic schemes and procedures can be found in the Supporting Information.

### MIC Determination

A single bacterial colony of *S. aureus* ATCC 29213 on an LB agar plate was inoculated into LB medium and grown overnight at 37 °C. The resulting culture was diluted 5000-fold and used as the inoculum to setup the MIC assay. **B1** was added to the growth medium in the first well of a 96-well plate. A 2-fold serial dilution was then generated from well 1 to 10. The last two wells were reserved for positive (without peptide) and negative (without bacteria) controls. Bacterial inoculum was supplemented with additives, as specified in the text (Ca­(II), PBA, or para-substituted PBAs) and then added to each well. The final volume in each well was 100 μL. The microtiter plate was incubated statically overnight at 37 °C prior to visual readout.

## Supplementary Material



## Notes

Extensive method descriptions, synthetic schemes, HPLC and HRMS analysis (PDF)
