# Supplementary material for: Mechanistic Studies of the Calcium-Dependent Antibiotics via Cofactor Engineering
Source: J Nat Prod. 2026 Jan 15;89(2):639–44. doi: 10.1021/acs.jnatprod.5c01440 (PMC12954748; doi:10.1021/acs.jnatprod.5c01440)
Supplement: Supplementary file 1 [file np5c01440_si_001.pdf]

SUPPORTING INFORMATION

Mechanistic Studies of the Calcium-Dependent Antibiotics via Cofactor Engineering

Shao-Lun Chiou[1§], Yu-Chi Chang[1§], Ya-Rong Chen, Thomas Ma, John Chu[1*]

[1] Department of Chemistry, National Taiwan University, No. 1 Sec. 4, Roosevelt Rd., Taipei 10617, Taiwan.

§ These authors contributed equally to this work

* Correspondence: johnchu@ntu.edu.tw

Table of Contents **Page**

Chemicals, instruments, and general methods	3
Solid-phase microwave-assisted peptide synthesis	3
Minimum inhibitory concentration determination	3
Preparation of N-(9-Fluorenylmethoxycarbonyl)-D-phenylglycine (Fmoc-D-Phg-OH)	4
Preparation of trifluoromethanesulfonic azide (TfN_3)	5
Preparation of (S)-2-azido-3-(tert-butoxy)propanoic acid (Azido-Ser(tBu)-OH)	5
**Scheme S1.** Preparation of FruB and its synthetic analog	6
**Scheme S2.** Preparation of CDAx and its synthetic analog	7
**Scheme S3.** Preparation of synthetic analog of Dap	9
**Figure S1.** CDAs structural alignment	11
**Figure S2.** ^{1}H and ^{13}C NMR spectra of Fmoc-D-Phg-OH	12
**Figure S3.** ^{1}H and ^{13}C NMR spectrum of N_3-Ser(tBu)-OH	13
**Figure S4.** HPLC and HRMS for FruB	14
**Figure S5.** HPLC and HRMS for $\mathbf{F_{Ser}}$	15
**Figure S6.** HPLC and HRMS of CDA4b	16
**Figure S7.** HPLC and HRMS of $\mathbf{C_{Ser}}$	16
**Figure S8.** HPLC and HRMS for $\mathbf{D_{Ser}}$	17
**Table S1.** List of *para*-substituted phenylboronic acids used in this manuscript	18
**Table S2.** MIC of **B1** and Hammett and the modified Swain-Lupton constants	18
**Table S3.** MIC of FruB and $\mathbf{F_{Ser}}$	19
**Table S4.** MIC of CDA4b and $\mathbf{C_{Ser}}$	19
**Table S5.** MIC of Dap and $\mathbf{D_{Ser}}$	20

Chemicals, instruments, and general methods

Source of boronic acids was listed in **Table S1**. Lysogeny broth (LB) was purchased from BioShop Canada Inc. Amino acid building blocks and coupling reagents were purchased from P3Biosystem, BLDpharm, and AgeneMax unless otherwise mentioned. Other chemicals were purchased from Merck, ThermoFisher Scientific, J.T.Baker, and Uni-Onward Corp. All chemicals are of ACS grade (or higher) and used as is. Peptides were purified using a C18 semi-preparative column (HYPER GLD AQ PREP, 5 μm, 250 × 10 mm, ThermoFisher Scientific) on a Waters HPLC Instrument (996 UV detector, 600 pump and controller) using a two-solvent gradient system, wherein solvent A is water with 0.1% (v/v) formic acid and solvent B is acetonitrile (ACN) with 0.1% (v/v) formic acid. All compounds are >95% pure by HPLC analysis. TLC mobility shift assays were performed on TLC Silica Gel 60 F254 (Merck). Mass spectra of synthetic peptides were acquired by using ESI-TOF (microTOF-QII, Bruker). All NMR spectra were acquired on a Bruker AVIII 400 (400 MHz) instrument.

Solid-phase microwave-assisted peptide synthesis

Biotage® Initiator+ Alstra™ was used for microwave-assisted coupling according to the manufacturer instruction. In each peptide coupling cycle, amino acid building blocks (5 equiv., 0.45 M in DMF), DIC (5 equiv., 0.5 M in DMF), and HOBt (5 equiv., 0.5 M in DMF) were added to the resin. The coupling reaction was performed using a peptide synthesizer with microwave assistance and oscillation at 75°C for 5 minutes. Fmoc protecting group was removed by 20% piperidine in DMF under room temperature (2 × 10 min).

Minimum inhibitory concentration determination

A single bacterial colony (*Staphylococcus aureus subsp. aureus*, ATCC 29213) on an LB agar plate was inoculated into LB medium (Bioshop®) and grown overnight at 37° C to stationary phase. The resulting culture was diluted 5,000-fold and used as the inoculum to setup the MIC assay. **B1** was added to the growth medium to generate the working solution (64 µg/mL, final concentrations ranged

from 32 to 0.0625 μg/mL), while other CDA analogues were prepared at a higher concentration (256 μg/mL, final concentrations ranged from 128 to 0.25 μg/mL), which was used to generate a 50 μL per well two-fold dilution series across a 96-well microtiter plate from well 1 to 10. The last two wells were reserved for positive (without peptide) and negative (without both peptide and bacteria) controls. Bacterial inoculum was mixed with specific concentration of additives (Ca(II), boronic acids) and then added to each well (50 μL), so that each well contained a total of 100 μL of solution. The microtiter plate was incubated statically at 37° C prior to visual readout.

Preparation of N-(9-Fluorenylmethoxycarbonyl)-D-phenylglycine (Fmoc-D-Phg-OH)

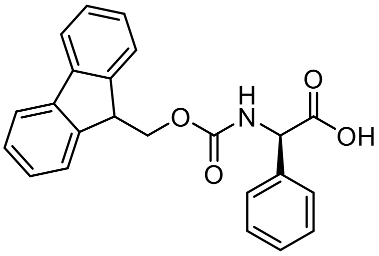

D-phenylglycine (150 mg, 1 mmol), K_2CO_3 (138 mg, 1 mmol) were dissolved in 5 mL of diH_2O/ACN (3:2). Fmoc-OSu (505 mg, 1.5 mmol) in 3 mL ACN was added. The reaction mixture was stirred at room temperature overnight. The organic solvent was removed under reduced pressure, and the remaining aqueous slurry was diluted with diH_2O. The solution was extracted with ether (3×) to remove by-products. The aqueous layer with desire product was further acidified to pH 2 with 1 M HCl and extracted with DCM (3×). The combined organic layers were dried over anhydrous $MgSO_4$, filtered, and concentrated under reduced pressure to afford a white solid (255 mg, 70% yield). TLC (DCM/MeOH/formic acid = 9/1/0.1) R_f = 0.3. ^{1}H NMR (400 MHz, DMSO-d_6) δ 8.18 (1H, d, J = 8.0 Hz), 7.88 (2H, d, J = 7.5 Hz), 7.76 (2H, d, J = 7.5 Hz), 7.45-7.28 (9H, m), 5.18 (1H, d, J = 8.0 Hz), 4.33-4.20 (3H, m); ^{13}C NMR (400 MHz, DMSO-d_6) δ 172.1, 155.9, 143.8, 140.7, 137.4 128.5, 127.9, 127.8, 127.7, 127.1, 125.4, 120.1, 66.0, 58.2, 46.7. ESI-HRMS calcd. m/z for $[C_{23}H_{19}NNaO_4]^+$ 396.1211, found 396.1206.

Preparation of trifluoromethanesulfonic azide (TfN_3)

Sodium azide (650 mg, 10 mmol) was dissolved in diH_2O (1.67 mL) and cooled in an ice bath. Trifluoromethanesulfonic anhydride (337 µL, 2 mmol) in DCM was added dropwise with stirring, and the mixture was stirred in the ice bath for 2 hours. The ice bath was then removed, and the reaction mixture was stirred at room temperature for another 2 hours. The product was extracted in DCM/H_2O. The DCM layer was separated using a separatory funnel, and the aqueous layer was further extracted with DCM (3 × 2 mL). The combined DCM extracts, containing trifyl azide (TfN_3), were washed once with saturated $NaHCO_{3(aq)}$. The resulting DCM solution of TfN_3 was used directly in subsequent reactions without further purification.

Preparation of (S)-2-azido-3-(tert-butoxy)propanoic acid (Azido-Ser(tBu)-OH)

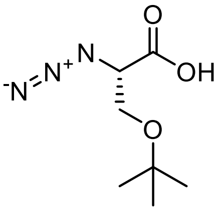

H-Ser(tBu)-OH (161 mg, 1 mmol), K_2CO_3 (207 mg, 1.5 mmol), and $CuSO_4 \cdot 5H_2O$ (2.5 mg, 0.01 mmol) were dissolved in 4 mL of diH_2O/MeOH (1:2). Triflic azide prepared in DCM was then added, and the reaction mixture was stirred at room temperature overnight. The organic solvent was removed under reduced pressure, and the remaining aqueous slurry was diluted with diH_2O and acidified to pH 6 with 1 M HCl. The solution was extracted with EA (3×) to remove by-products. The aqueous layer with desire product was further acidified to pH 2 with 1 M HCl and extracted with EA (3×). The combined organic layers were dried over anhydrous $MgSO_4$, filtered, and concentrated under reduced pressure to afford a yellowish oil (112 mg, 60% yield) without further purification. TLC (DCM/MeOH/formic acid = 9/1/0.1) R_f = 0.4. ^1H NMR (400 MHz, $CDCl_3$) δ 10.69 (1H, br), 3.95 (1H, t, *J* = 4.5 Hz), 3.82 (1H, d, *J* = 4.8 Hz), 3.82 (1H, d, *J* = 4.2 Hz), 1.21 (9H, s); ^{13}C NMR (400 MHz, $CDCl_3$) δ 174.4, 74.6, 62.8, 61.5, 27.2. ESI-HRMS calcd. m/z for $[C_7H_{13}N_3NaO_3]^+$ 210.0849, found 210.0851.

Preparation of FruB and its synthetic analogs

Peptide synthesis was initiated from Asp5. 2-CTC resin was swollen in DCM in a RV at room temperature for 30 minutes. After removing the solvent, a solution of Fmoc-Asp(tBu)-OH (2 equiv.) and DIPEA (4 equiv.) in DCM was added to the resin. The mixture was agitated on an orbital shaker (180 rpm) at room temperature for 16 hours. The loading solution was then removed, and the resin was washed with DCM (5×). To cap unreacted linkers on the resin, a capping solution (DCM/MeOH/DIPEA = 17/2/1, v/v) was added to the RV and the resin was agitated at room temperature for 30 minutes. The resin was then washed with DCM (3×) and DMF (3×). Loading efficiency was determined by the standard procedure according to UV absorbance from Fmoc deprotection (0.6 mmol/g).

The peptide was then synthesized from Asp4 to the N-terminal palmitic acid by microwave-assisted peptide synthesizer. The alloc group on Dap2 was deprotected by a solution of $Pd(PPh_3)_4$ (0.2 equiv.) and $PhSiH_3$ (10 equiv.) in DCM[a]. The mixture was agitated under argon at room temperature, and the reaction was performed twice, each for 1 hour, using freshly prepared reagents. After the reaction, the resin was washed with DCM (3×) and DMF (3×). The peptide was then synthesized from Pro11 to Gly6 by microwave-assisted peptide synthesizer. After the final building block was coupled, 10 mL 1% TFA in DCM was added to the RV. The mixture was left standing at room temperature for 10 minutes. The solution was then collected into a round-bottom flask and diluted by DCM to 100 mL. To this solution, DIC (10 equiv.) and HOBt (10 equiv.) were added, and the mixture was stirred at room temperature for 2 days[b].

The solution was concentrated under reduced pressure. Then, 10 mL of TFA/TIPS/H_2O (95/2.5/2.5, v/v) was added for global deprotection[c]. The mixture was left standing at room temperature for 1 hours. After deprotection, TFA was evaporated under reduced pressure. Then 2 mL H_2O was added and the mixture was then purified by reversed-phase HPLC using a semi-preparative column. The peptide was eluted at around 67% B. Peptide-containing fractions were pooled, concentrated under reduced

pressure, and lyophilized. The final product was stored as a dry powder at -20 °C until use. The yield was about 2.0% based on resin loading.

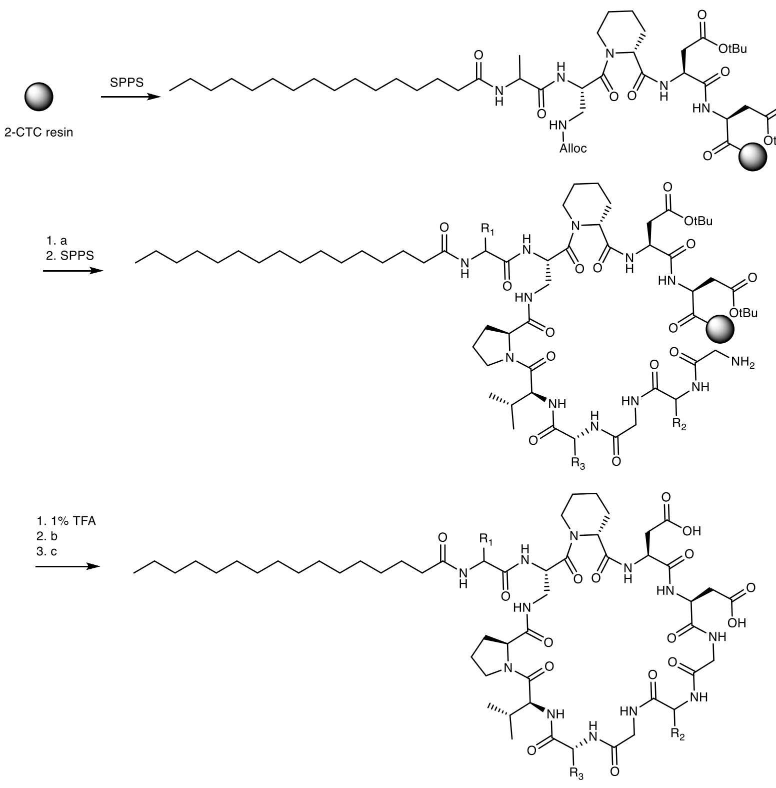

Scheme S1. Synthesis of FruB analogues. a. $Pd(PPh_3)_4/PhSiH_3$, b. DIC/HOBt, c. $TFA/H_2O/TIPS$ (95:2.5:2.5, v/v)

Synthesis of CDAx and its synthetic analogs

Peptide synthesis was initiated from the C-terminal residue, Trp11. 2-CTC resin was swollen in DCM in a RV at room temperature for 30 minutes. After removing the solvent, a solution of Fmoc-Trp(Boc)-OH (2 equiv.) and DIPEA (4 equiv.) in DCM was added to the resin. The mixture was agitated on an orbital shaker at room temperature for 16 hours. The loading solution was then removed, and the resin was washed with DCM (5×). To cap unreacted linkers on the resin, a capping solution (DCM/MeOH/DIPEA = 17/2/1, v/v) was added to the RV and the resin was agitated at room temperature for 30 minutes. The resin was then washed with DCM (3×) and DMF (3×). Loading

efficiency was determined by the standard procedure (0.8 mmol/g).

The remaining sequence (AA10 to AA1, followed by decanoic acid) was synthesized using standard coupling procedures. For Thr2, Fmoc-Thr-OH without side chain protection was used. After coupling the final building block, 10 mL 1% TFA in DCM was added to the RV. The mixture was left standing at room temperature for 10 minutes. The solution was the collected into a round-bottomed flask and diluted with DCM. To this solution, DIC (10 equiv.) and DMAP (1 equiv.) were added, and the mixture was stirred at room temperature for 3 days[d].

After the reaction, the solution was concentrated under reduced pressure. Then, 10 mL of TFA/TIPS/H_2O (95/2.5/2.5, v/v) was added for global deprotection[e]. The mixture was left standing at room temperature for 2 hours. After deprotection, TFA was partially evaporated under a stream of air. Then, 20 mL of cold ether (pre-chilled to -20℃) was added to precipitate the peptide. After centrifugation, the supernatant was removed. The pelleted peptide was redissolved in H_2O/ACN (1/1, v/v). The solution was then purified by reversed-phase HPLC using a semi-preparative column. Peptide-containing fractions at around 60% B were pooled, concentrated under reduced pressure, and lyophilized. The final product was stored as a dry powder at -20 °C until use. The yield was around 1.1% based on resin loading.

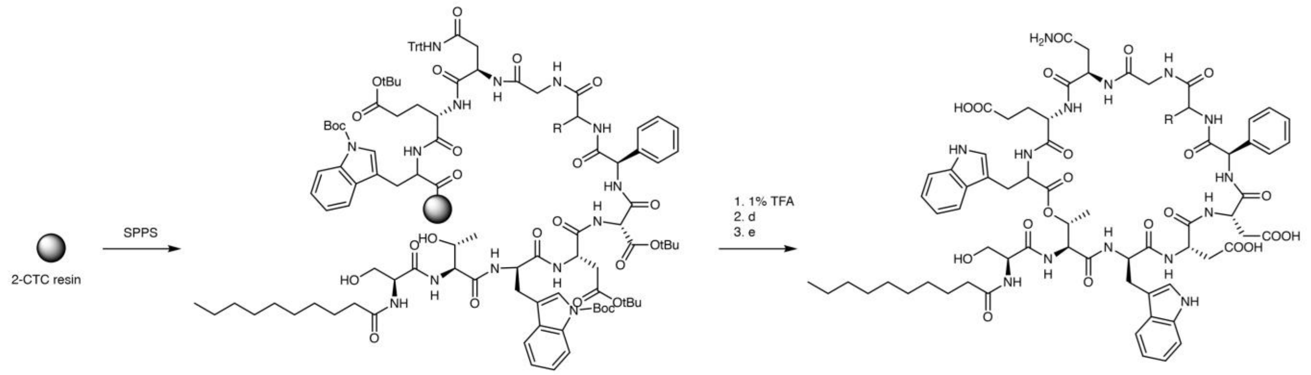

Scheme S2. Synthesis of CDA4b analogues. d. DIC/DMAP, e. TFA/H_2O/TIPS (95:2.5:2.5, v/v)

Synthesis of Dap and its synthetic analogues

Peptide synthesis was initiated by loading Asp7 onto Wang resin. The resin was swollen in DCM in a RV for 30 minutes. After removing the solvent, a solution of Fmoc-Asp-OAll (5 equiv.), DIC (5 equiv.), and DMAP (0.5 equiv.) in DCM was added to the resin. The mixture was agitated on an orbital shaker at room temperature for 16 hours. The loading solution was then removed, and the resin was washed with DCM (5×). To cap unreacted linkers on the resin, a capping solution (Ac_2O/DIPEA/DCM = 9/1/0.1, v/v) was added to the RV and the resin was agitated at room temperature for 1 hour. The resin was then washed with DCM (3×) and DMF (3×). Loading efficiency was determined by the standard procedure (0.4 mmol/g).

Residues AA6 to Ser3 (coupled as N_3-Ser(tBu)-OH) were assembled by sequential coupling, using peptide synthesizer. After coupling of the Ser3 residue, the resin was washed with DCM (5×). A solution of Fmoc-Trp(Boc)-OH (5 equiv.), DIC (5 equiv.), DMAP (5 equiv.) in DCM was then added to the resin[f]. The mixture was agitated at room temperature for 24 hours to enable esterification. After the reaction, the resin was washed with DCM (3×) and DMF (3×). The sequence from Glu12 to D-Ala8 was then assembled using standard coupling procedures. After coupling of the D-Ala8 residue, the resin was washed with DCM (5×). For allyl group deprotection, a solution of $Pd(PPh_3)_4$ (0.2 equiv.) and $PhSiH_3$ (10 equiv.) in DCM was added to the resin[g]. The mixture was agitated under argon at room temperature, and the reaction was performed twice, each for 1 hour, using freshly prepared reagents. After the reaction, the resin was washed with DCM (3×) and DMF (3×). The Fmoc protecting group was then removed[h]. After deprotection, a solution of HATU (3 equiv.) and DIPEA (6 equiv.) in DMF was added to the resin[h]. The mixture was agitated at room temperature for 2 hours. After the reaction, the resin was washed with DMF (5×). A solution of dithiothreitol (DTT, 2 M) and DIPEA (1 M) in 2 mL of DMF was then added to the resin to deprotect azido protecting group on Ser3[i]. The mixture was agitated at room temperature, and the reaction was performed twice, each for 2 hours, using freshly prepared reagents. The sequence from D-Asn2 to the N-terminal decanoic acid was then assembled by peptide synthesizer.

After coupling the final building block, the resin was washed with DCM (5×). TFA/TIPS/H_2O (5 mL, 95/2.5/2.5, v/v) was added to the RV, and the mixture was left at room temperature for 2 hours for global deprotection and cleavage[j]. The cleavage solution was the collected, and TFA was partially evaporated under a stream of air. Then, 20 mL of cold ether (pre-chilled to -20 °C) was added to precipitate the peptide. After centrifugation, the supernatant was removed. The pelleted peptide was redissolved in H_2O/ACN (1/1, v/v) and the solution was purified by reversed-phase HPLC using a semi-preparative column. Peptide-containing fractions at around 55% B were pooled, concentrated under reduced pressure, and lyophilized. The final product was stored as a dry powder at -20 °C until use. The yield was around 1.2% based on resin loading.

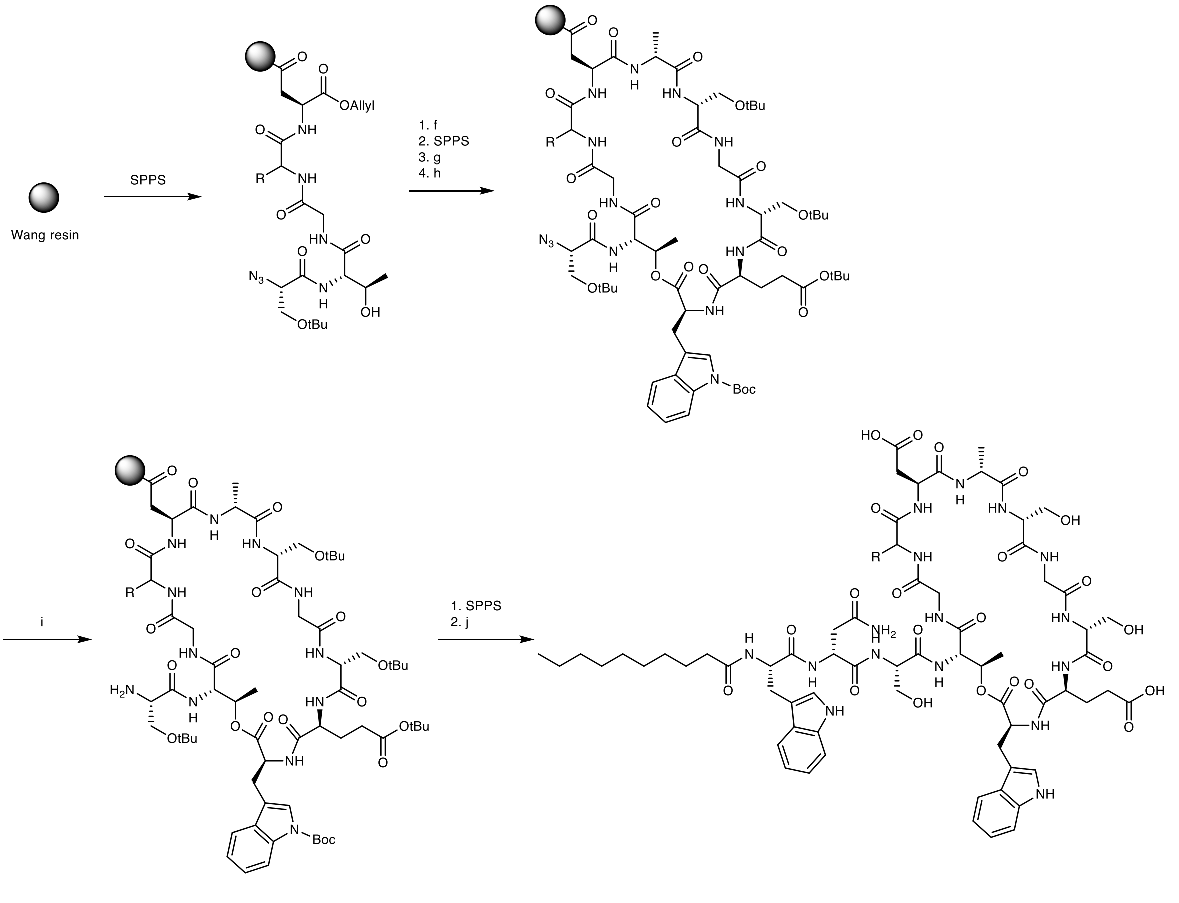

Scheme S3. Synthesis of daptomycin analogue. f. DIC/DMAP/Fmoc-Trp-OH, g. $Pd(PPh_3)_4/PhSiH_3$, h. DIC/HOBt, i. DTT/DIEA, j. TFA/H_2O/TIPS (95:2.5:2.5, v/v)

Figure S1. CDAs structural alignment.

Upper case and lower case of the three-letter code represent l- and d-amino acids, respectively. Acidic and Basic residues were marked in red and blue, respectively. Minor side chain modifications were underlined. CDA marked in red dots were used in this study.

Name	FA	Exocyclic part				Macrocycle									
		P-4	P-3	P-2	P-1	P1	P2	P3	P4	P5	P6	P7	P8	P9	P10
● Daptomycin	FA		Trp	asn	Asp	**Thr**	Gly	Orn	Asp	ala	Asp	Gly	ser	Glu	Kyn
Taromycin	FA		Trp	asn	Asp	**Thr**	Gly	Orn	Asp	ala	Asp	Gly	ala	Glu	Kyn
A54145	FA		Trp	glu	Asn	**Thr**	Gly	Ala	Asp	lys	Asp	Gly	asn	Glu	Ile
● CDAx	FA				Ser	**Thr**	trp	Asp	Asp	hpg	Asp	Gly	asn	Glu	Trp
● Laspartomycin	FA				Asp	**Dap**	pip	Gly	Asp	Gly	Asp	Gly	thr	Ile	Pro
Amphomycin	FA				Asp	**Dap**	pip	Asp	Asp	Gly	Asp	Gly	dap	Val	Pro
● Friulimicin	FA				Asn	**Dap**	pip	Asp	Asp	Gly	Asp	Gly	dap	Val	Pro
Cadaside	FA		Ala	glu	Tyr	**Thr**	ile	Asp	Asp	Pro	Gly	glu	Glu	Gly	
Malacidin	FA				Asp	**Dap**	val	Lys	Asp	Asp	Gly	asp	Val	Pro	
Ambocidin	FA	Gly	Gly	ser	Tyr	Thr	ile	Arg	Gly	Asp	Gly	Gly	Thr	Hpg	

Figure S2. NMR spectrum of Fmoc-D-Phg-OH

a. ^{1}H NMR spectrum of Fmoc-D-Phg-OH (400MHz, DMSO-d_6)

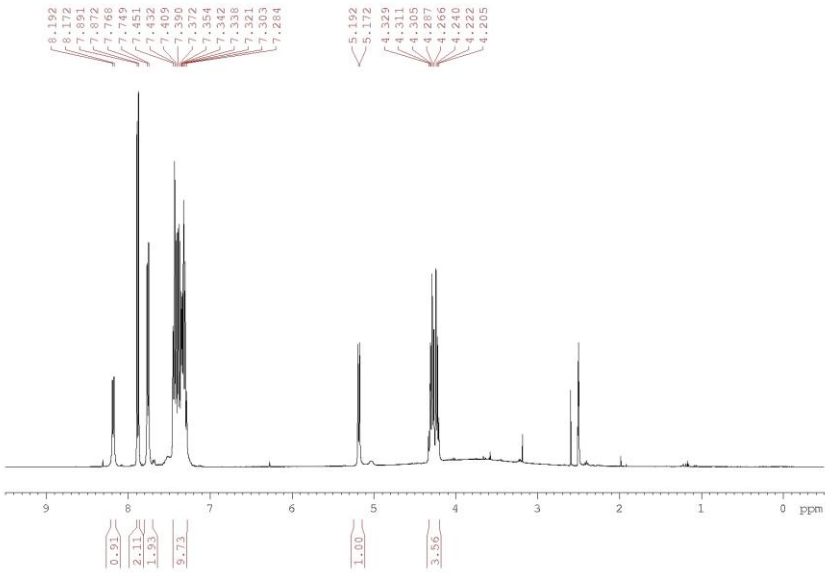

b. ^{13}C NMR spectrum of Fmoc-D-Phg-OH (400MHz, DMSO-d_6)

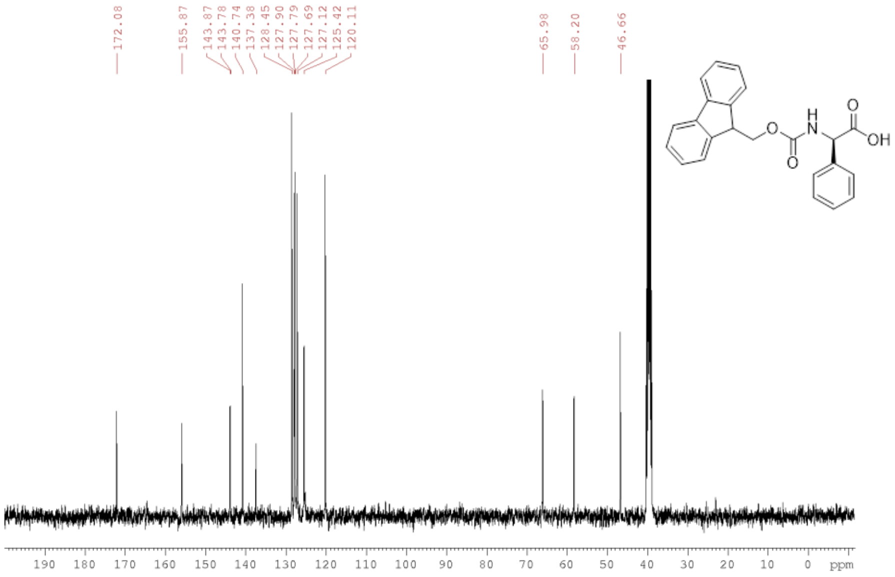

Figure S3. NMR spectrum of N_3-Ser(tBu)-OH

a. ^1H NMR spectrum of N_3-Ser(tBu)-OH (400MHz, $CDCl_3$)

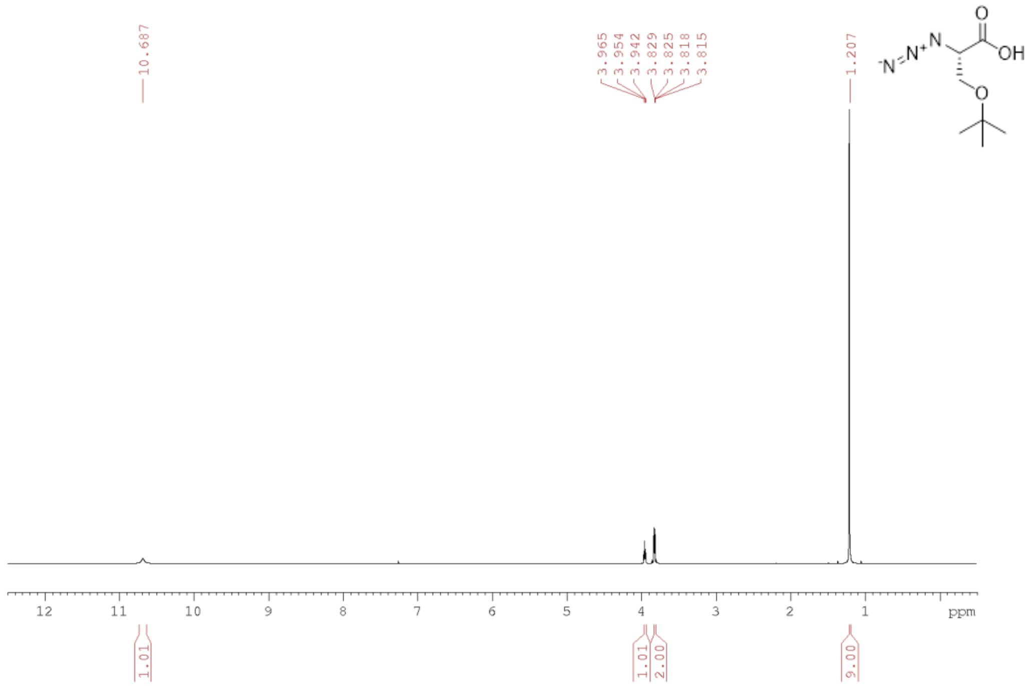

b. ^{13}C NMR spectrum of N_3-Ser(tBu)-OH (400MHz, $CDCl_3$)

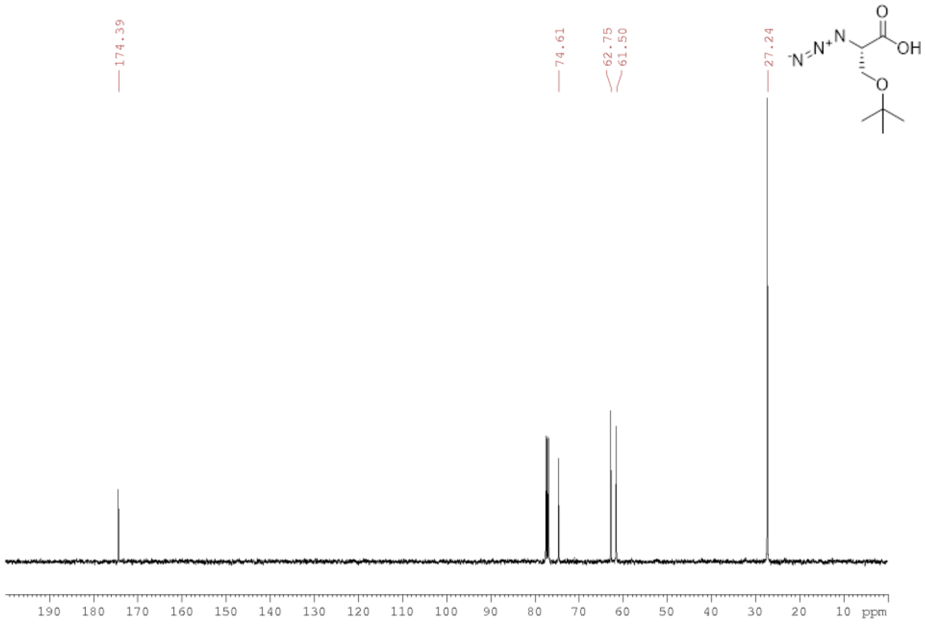

Figure S4. HPLC and HRMS for FruB

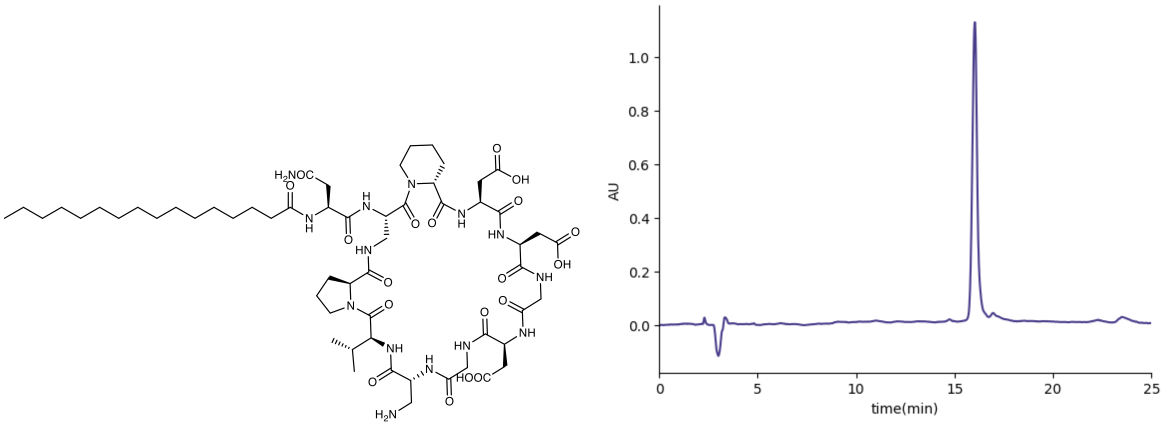

ESI-HRMS calcd. m/z for $[C_{58}H_{94}N_{14}O_{19}]^+$ 1291.6901, found 1291.6883

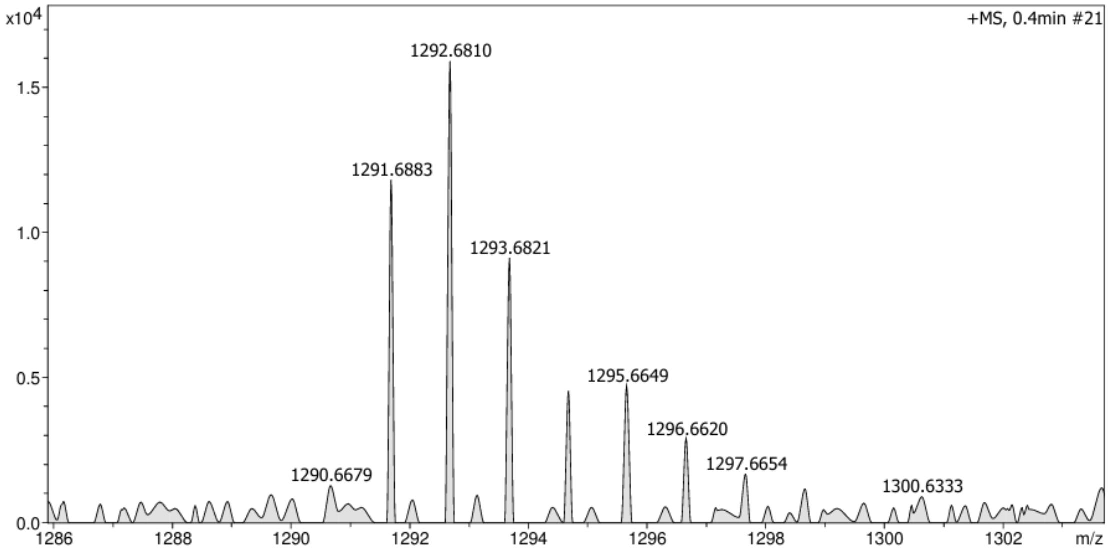

Figure S5. HPLC and HRMS for $\mathbf{F_{Ser}}$

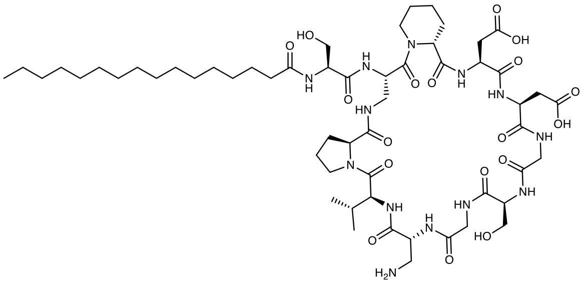

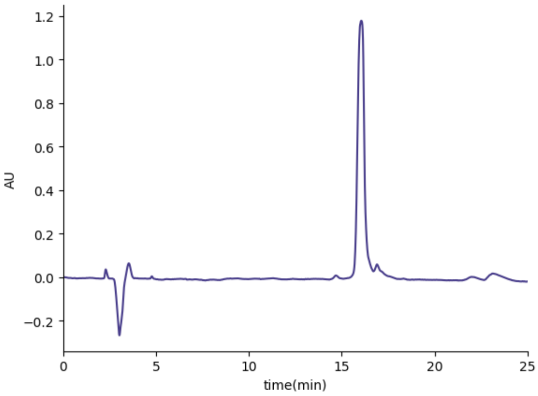

ESI-HRMS calcd. m/z for $[C_{56}H_{93}N_{13}O_{18}]^+$ 1236.6843, found 1236.6871

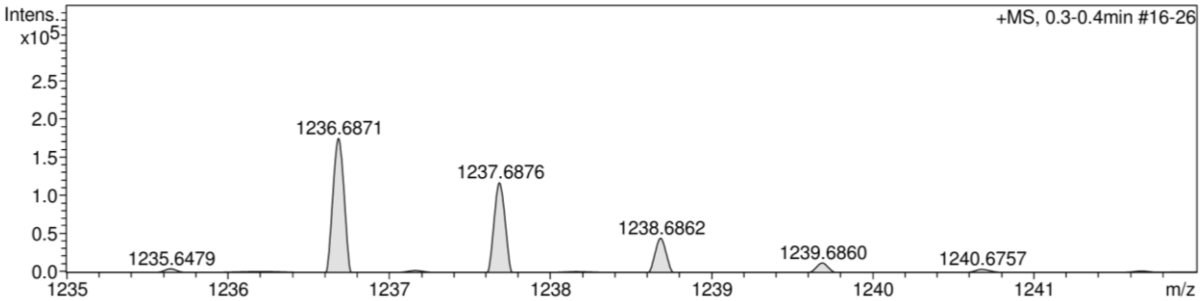

Figure S6. HPLC and HRMS for CDA4b

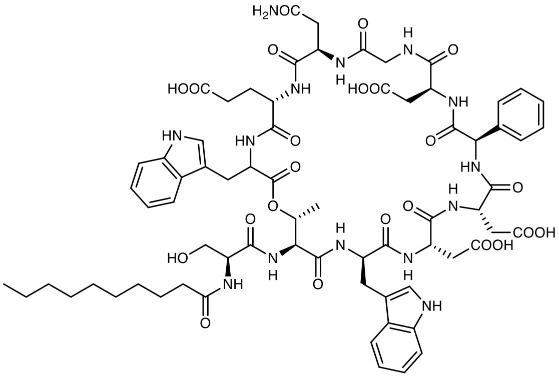

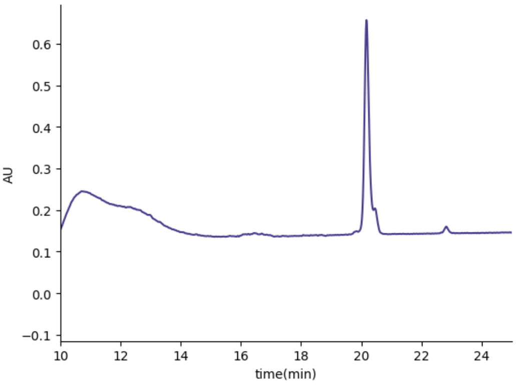

ESI-HRMS calcd. m/z for $[C_{70}H_{89}N_{14}O_{23}]^+$ 1493.6219, found 1493.6249

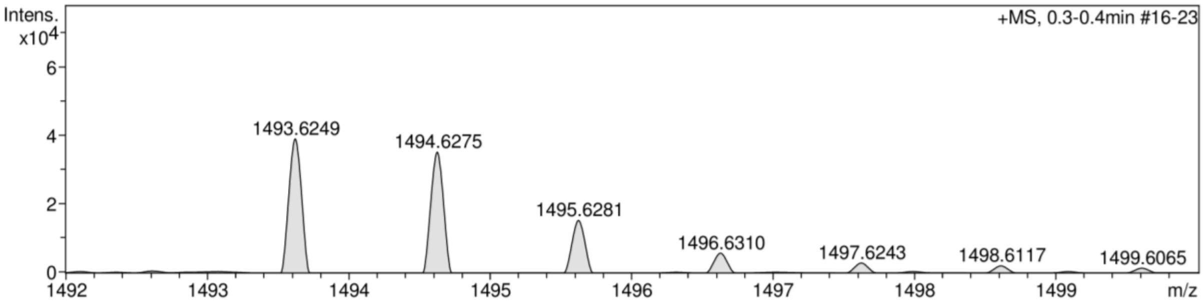

Figure S7. HPLC and HRMS for $\mathbf{C_{Ser}}$

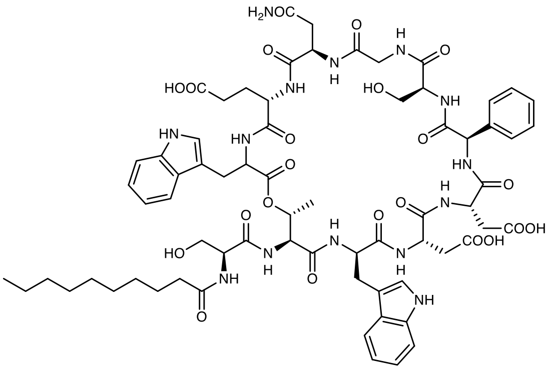

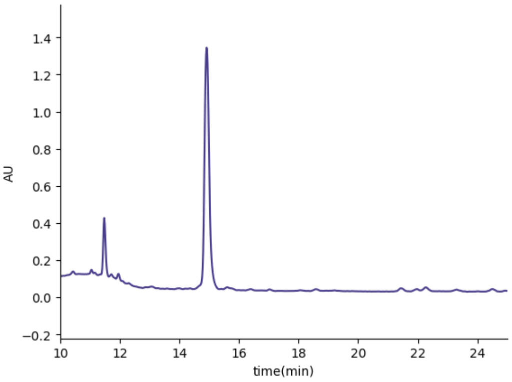

ESI-HRMS calcd. m/z for $[C_{69}H_{89}N_{14}O_{22}]^+$ 1465.6270, found 1465.6284

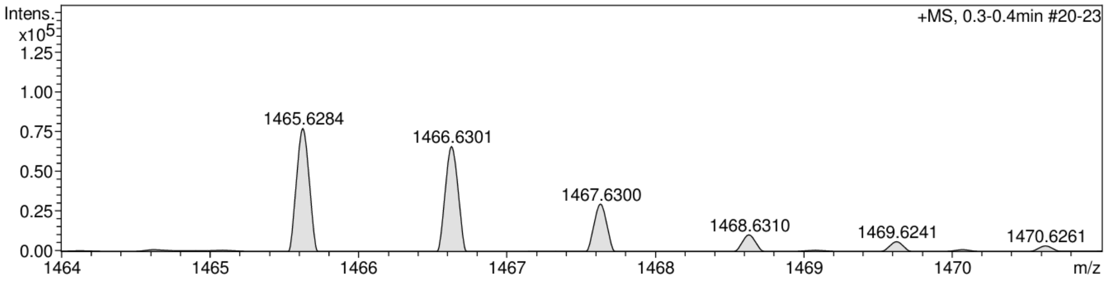

Figure S8. HPLC and HRMS for **D_{Ser}**

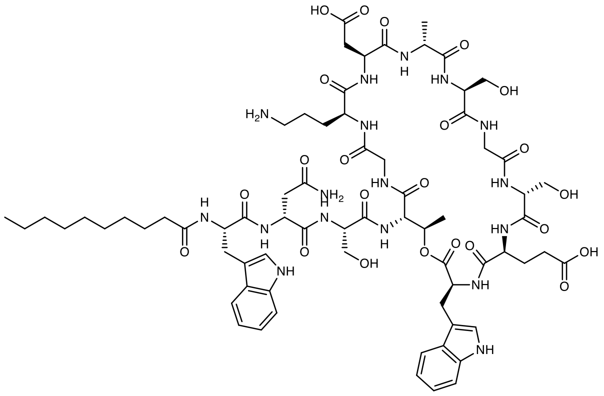

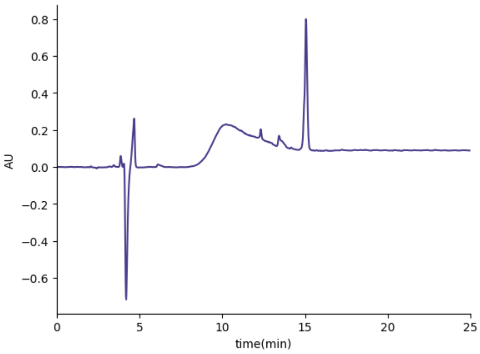

ESI-HRMS calcd. m/z for $[C_{70}H_{100}N_{17}O_{23}]^+$ 1546.7172, found 1546.7127

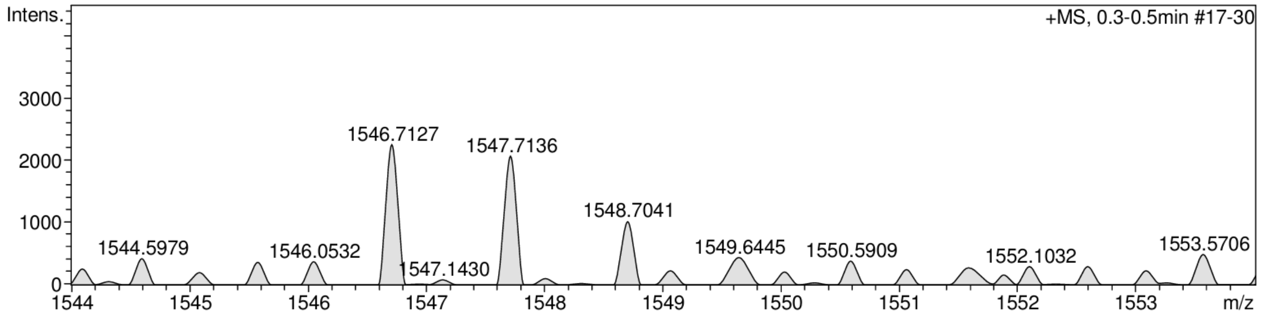

Table S1. List of boronic acids used in this manuscript

Compound	Supplier
4-hydroxyphenylboronic acid	Aldrich
4-aminophenylboronic acid hydrochloride	Combi-Blocks
4-cyanophenylboronic acid	Matrix
4-methoxyphenylboronic acid	Acros
4-pyridylboronic acid	HyBio
4-fluorophenylboronic acid	BLD pharm
4-(methylthio)phenylboronic acid	Acros
4-tolylboronic acid	AK Scientific
4-chlorophenylboronic acid	BLD pharm
4-bromophenylboronic acid	BLD pharm
4-nitrophenylboronic acid	BLD pharm

Table S2. MIC of **B1** and Hammett and modified Swain-Lupton Constants (σ, F, R) of *p*-boronic acids. F and R are constants that contribute to inductive and resonance effect of Hammett constant, respectively.

PBA derivative	MIC	$\log_2$(MIC)	σ	F	R
**p-nitro**	4	2	0.78	0.65	0.13
**p-methoxy**	8	3	-0.27	0.29	-0.56
**p-fluoro**	8	3	0.06	0.45	-0.39
**p-cyano**	8	3	0.66	0.51	0.15
**p-chloro**	8	3	0.23	0.42	-0.19
**p-bromo**	8	3	0.23	0.45	-0.22
**p-thiomethyl**	16	4	0	0.23	-0.23
**p-hydroxy**	16	4	-0.37	0.33	-0.7
**p-amine**	16	4	-0.66	0.08	-0.74
**p-methyl**	32	5	-0.17	0.01	-0.18

Table S3. MIC of FruB and **F_{Ser}**. “>” denotes MIC greater than 128 μg/mL.

FruB		PBA (mg/mL)	
		0.1	0
Ca(II) (mM)	5	4	8
	0	>	>

**F_{Ser}**		PBA (mg/mL)	
		0.1	0
Ca(II) (mM)	5	>	>
	0	>	>

Table S4. MIC of CDA4b and **C_{Ser}**. “>” denotes MIC greater than 128 μg/mL. “-“ denotes not determined.

CDA4b		PBA (mg/mL)			
		0.1	0.05	0.025	0
Ca(II) (mM)	64	32	-	-	-
	16	32	32	32	64
	4	>	-	-	-
	0	>	-	-	>

**C_{Ser}**		PBA (mg/mL)			
		0.1	0.05	0.025	0
Ca(II) (mM)	64	16	-	-	-
	16	32	64	128	128
	4	128	-	-	-
	0	128	-	-	>

Table S5. MIC of Dap and **D$_{Ser}$.** “>” denotes MIC greater than 128 μg/mL. *The parenthesized result was determined in 0.125 mM EDTA which is sufficient to quench the effect of Ca(II) in LB broth.

Dap		PBA (mg/mL)	
		0.1	0
Ca(II) (mM)	1.25	0.5	0.5
	0	16(>)*	16(>)*

**D$_{Ser}$**		PBA (mg/mL)	
		0.1	0
Ca(II) (mM)	1.25	>	>
	0	>	>
